# Embracing Integrative Multiomics Approaches

**DOI:** 10.1155/2016/1715985

**Published:** 2016-09-04

**Authors:** Daniel M. Rotroff, Alison A. Motsinger-Reif

**Affiliations:** ^1^Bioinformatics Research Center, North Carolina State University, Raleigh, NC 27607, USA; ^2^Department of Statistics, North Carolina State University, Raleigh, NC 27607, USA

## Abstract

As “-omics” data technology advances and becomes more readily accessible to address complex biological questions, increasing amount of cross “-omics” dataset is inspiring the use and development of integrative bioinformatics analysis. In the current review, we discuss multiple options for integrating data across “-omes” for a range of study designs. We discuss established methods for such analysis and point the reader to in-depth discussions for the various topics. Additionally, we discuss challenges and new directions in the area.

## 1. Introduction

The past decade has witnessed tremendous advancements in biotechnology and computational performance that have provided vast amounts of new data and accompanying optimism for the burgeoning improvements to human health and disease treatment. It is now possible, and increasingly routine, for studies to test thousands to millions of molecular endpoints. However, as the dimensionality of these data increases, larger sample sizes are required, and studies are now being conducted on an unprecedented scale. We are now seeing an explosion of data in almost every area of disease and clinical research. These technologies are commonly referred to as “omics” technologies, related to the suffix “-ome,” defined as “all constituents considered collectively” [1]. We now have vast amounts of data related to the genome, transcriptome, epigenome, proteome, and metabolome. In fact, many of these areas of research have spawned subfields that are rapidly advancing our mechanistic understanding of biology (e.g., pharmacogenomics, metagenomics, lipidomics, kinomics, and secretomics). However, too often we as researchers find small amounts of variation explained and are left with “missing heritability” and unexplained variation, reinforcing the seemingly exponential complexity of biology [2]. It seems as Robert M. Persig said that “the number of rational hypotheses that can explain any given phenomenon is infinite” [3]. Although it is clear that no single “-omics” technology can fully capture the intricacy of most complex diseases or other clinically relevant traits, the collective information from each of these technology platforms when combined has the potential to offer incredible insight into the mechanisms of complex disease and other important clinical traits.

Although it is clear that data integration is required, methods for achieving this are far from systematic. The integrative genomics methodologies that are used to interpret these data require expertise in multiple different disciplines, such as biology, medicine, mathematics, statistics, and bioinformatics. Such interdisciplinary approaches require diverse expertise, either through extensive interdisciplinary training or through extensive collaborations. The accumulation of enormous quantities of molecular data has led to the emergence of “systems biology”—a branch of science that discovers the principles that underlie the basic functional properties of living organisms, starting from interactions between macromolecules. Integrative genomics is based on the fundamental principle that any biological mechanism builds upon multiple molecular phenomena, and only through the understanding of the interplay within and between different layers of genomic structures can one attempt to fully understand phenotypic traits. Therefore, principles of integrative genomics are based on the study of molecular events at different levels and on the attempt to integrate their effects in a functional or causal framework.

## 2. Tools for Integrative Analysis

### 2.1. Using Publically Available Databases

Commonly used approaches involve linking all markers at the genomic, proteomic, metabolomics, and other levels back to annotated genes. In general, this approach works sufficiently because well annotated and curated databases describing genes and their known biological functions are readily available, though the various sources of data can be a challenge for analysis. Examples of these databases include NCBI's gene database (http://www.ncbi.nlm.nih.gov/gene/), gene ontology (GO) (http://geneontology.org/), Ensembl (http://useast.ensembl.org), KEGG (http://www.genome.jp/kegg/pathway.html), HMDB (http://www.hmdb.ca/), MetaCyc (http://metacyc.org/), WikiPathways (http://www.wikipathways.org/index.php/WikiPathways), and DAVID (http://david.abcc.ncifcrf.gov/), and many others are also available. For data that is more granular than the “gene level” (e.g., SNPs, CpGs), methods for combining dependent univariate test statistics or *p* values are now available (e.g., SKAT [4], Correlated Lancaster Approach [5], and decorrelation tests [6]). As an example, the Correlated Lancaster Approach is a modified version of the Fisher method for combining multiple *p* values; however, when *p* values are correlated the Fisher method for combining *p* values will cause inflation of Type I error rates [5]. The Correlated Lancaster Approach addresses this by accounting for the underlying correlation structure of *p* values to limit Type I error and allowing for *p* values from multiple tests to be aggregated appropriately [5]. Now that resources such as 1000 genomes (http://www.1000genomes.org/) are available, methods for genotype imputation [7, 8] have made it possible to merge different genotyping platforms therefore greatly enhancing the ability to integrate genomics data and perform meta-analyses.

However, some data types are not readily mapped to annotated genes and these annotation limitations are particularly noticeable for the newest “omics” technologies. Metabolomics, for example, has major gaps in annotation that limit integration potential and limit the utility of pathway based and integrative methods approaches [9]. Metabolomics data is typically interpreted in the context of metabolic pathways and KEGG is an example of a database that contains metabolic pathways consisting of both metabolites and enzymes organized into groups related to metabolism, cellular processes, human diseases, and others. However, the lack of annotated metabolites indicates that we still have much to learn about the role of many metabolites in human health. Improved understanding of how genetic variants affect downstream molecular changes, such as metabolite levels, will be critical to improving our ability to interpret and integrate these types of data.

Once the results are mapped to annotations in a database, various integrative analysis approaches can be taken. While “integrative analysis” and “systems biology” can be vaguely defined workflows, in the current discussion we will consider the analysis of at least two different types of omics data as integrative.

The analysis can be restricted to molecular data (such as in expression quantitative trait loci (eQTL) studies, in which the relation between germ line variation and gene expression is investigated) or it can involve clinical outcomes (e.g., disease status or treatment response) or intermediate phenotypes and biomarkers.

### 2.2. Selecting the Appropriate Analysis Strategy

While it is possible to design an analysis plan to ask a variety of interesting biological and clinical questions, there are a few themes that usually emerge. The first common objective of analysis is to understand molecular behaviors, mechanisms, and relationships between and within the different types of molecular structures, including associations between these and various phenotypes, such as clinical outcomes and pathways. The second objective is often to understand the taxonomy of diseases or other clinical traits, thereby classifying individuals into latent classes of disease subtype; and the third objective is to predict an outcome or phenotype for prospective patients. Some statistical methods are specialized to one type of question, and others can be used for several. Some of the tools, such as enrichment analysis, were originally designed to reveal features of genes and pathways, whereas others, such as integrative clustering, were designed to reveal features of patient subgroups; however, most of the tools discussed below can be applied to both. The statistical methods used can be unsupervised or supervised (e.g., according to whether one proceeds in an exploratory manner or applies clinical labels to individual cases). Often these methods are used in conjunction with cross-validation or other model selection approaches to prevent overfitting.

### 2.3. Sequential Analysis

One of the most commonly used approaches, because of its ready application and interpretation, is sequential analysis. In sequential analysis, evidences (measures of association, etc.) from distinct omics levels are used. This approach allows the confirmation or refinement of findings based on one data type, with additional analyses of further omics data obtained from the same set of samples. In this case, at least two types of omics data are analyzed, for example, copy-number variants (CNVs) and gene expression level data.

Typically, in sequential approaches, an analysis of each dataset is made independently of the others and produces a list of interesting entities (omics level variables), which are then linked to each other. For example, differentially expressed genes in one list are compared with each other and then with different CNVs that have been matched to the closest gene in a second list. Usually, the lists are intersected to find the genes that are confirmed in the analysis of each data type. Comparing ranks of each gene in each list leads to measures of concurrence. If each entity in each list has a value of association with the outcome of interest (e.g., a *t*-test statistic) then these values can be combined, though there are challenges in how to create a combined *p* value to this intersection after proper controls for multiple comparison. Approaches for combining *p* values and permutation testing are suggested approaches.

Occasionally, the various analyses are not performed in parallel but as a sequence of filtering steps, each functioning on a single data type. This approach can simplify statistical inference, but the results are highly dependent on ordering of the steps in the sequence. Such differences can be difficult to interpret and add a layer of complexity to ensuring reproducibility of analysis. Because of this, details of methods, including annotation details, must be shared at the level of databases and code to ensure reproducibility.

### 2.4. Gene Set and Pathway Based Analyses

Another very important area of integrative analysis is gene set/pathway analysis. These approaches integrate biological knowledge across omics levels through expert driven and computationally derived knowledge bases. This is a way to perform integrative analysis even when only a single omics level has been collected for a particular dataset. The knowledge bases incorporate and integrate data from a variety of omics levels to aid in systematic understanding. Pathway analysis methods can test whether the effects observed are enriched for various biological functions. These methods range in both complexity of statistical methods and the level of detail required to conduct the analysis. Relatively simple approaches, such as overrepresentation analyses (ORA), only require a set of statistically significant endpoints (e.g., genes, metabolites, and proteins) that test for enrichment in a set of endpoints known to be related to a biological process [10]. Slightly more complex approaches, such as GSEA [11] or the Correlated Lancaster Approach [5], use all of the data as either ranks or test statistics to determine if enrichment exists. These methods use all available data, addressing the limitation of ORA approaches which rely on an arbitrary significance threshold. Since many available databases contain more information than just groups of endpoints (e.g., genes, metabolites), incorporating information, such as pathway topology, will ultimately be desirable. Although the best way to incorporate these relationships is still an active research area, some methods (i.e., impact factor analysis) are currently able to leverage this information [10]. A full discussion of pathway and gene set analysis methods is beyond the scope of the current paper; an excellent review of the methods commonly used for this type of analysis can be found in [10].

### 2.5. Replication as a Form of Integration

Methods development is an incredibly active area of research, and promising new methods for integrating “-omics” data are on the horizon. The simplest of these approaches use different “-omics” technologies as “pseudo replication” across “-omes,” building on the simple overlap approaches discussed above. Specifically, sophisticated Bayesian approaches incorporate information from one “ome” as prior information to perform association analysis for other, distinct “omes.” Additionally, there are a number of clustering and network based analysis tools that do not rely on established knowledge bases and have the potential to discover new biology. However, these approaches have not been widely used due to the limited number of datasets amenable to such analysis [12, 13]. An excellent review of newer approaches for “omics” integration is included in Ge et al. [14].

### 2.6. Constantly Evolving Methods

Although methods and data integration techniques are continuously evolving, there are several challenges that will need to be addressed in order for an integrative approach to become standardized and routine. From a statistical perspective, the most fundamental challenge in integrative analyses is dimensionality: taking more levels into account in the analysis tends to increase the dimensionality of the problem. Adding more layers of data or increasing the resolution of measurements increases the dimension of unknown parameters, which are often difficult to estimate, thereby making the overall inference weaker. This might seem paradoxical, as the purpose of taking multiple levels into account is precisely the opposite—to use more observations to obtain a more accurate picture of the biological system under study. In addition to the challenges described above with high dimensional data, a formidable quandary is how best to link data across omics platforms and different levels of biology. These relationships often produce “one-to-many” relationships, making causal relationships difficult to define.

There are also a number of limitations in the data curation and quality control. In addition, at every step, there will be checkpoints of compatibility of the data, such as normalization to the same scale, sample selection from representative cohorts, adequate correction for technical batch effects, and use of different platforms. Constantly evolving technologies exacerbate this challenge.

Additionally, the variety of study designs underlying individual “-omics” datasets poses a significant problem to integrating data across multiple studies. Many studies are cross-sectional and only capture a snapshot of what is actually a highly dynamic system (e.g., transcriptomics). In addition, cohort differences due to individual study goals or available study populations may pose a significant barrier for integrating data. Even if the study designs are similar, different technology platforms have varying resolutions. Although genotype imputation has reduced this barrier in genome-wide association studies, a comparable tool does not exist for most “-omics” platforms. Pathway databases are continuously improving, but currently information related to tissue type, cell type, developmental stage (young, old), and diseased state is extremely sparse. We need methodological improvements that can address pathway topologies and feedback loops and methods to simulate data to benchmark pathway analysis methods.

### 2.7. Comparative Approaches for Integrating Omics Data

Another aspect of data integration and “omics” integration can be seen in the growing reliance on in vitro model systems and comparative genetics approaches with model organisms of disease. To date, there have been many successful examples of comparative genomics that implement a multiomics strategy to validate or replicate signals. For example, lymphoblastoid cell line models have shown success in finding gene and gene expression results that support clinical genetic results [15]. Results across species, such as domestic dogs, have also been shown to be excellent approaches for omics integration [16, p. 1], [17]. Cancer research is an example where a comparative approach using canines holds particular promise [18]. Comparisons of DNA copy-number aberrations in canines and human have provided valuable insight into the mechanisms of osteosarcoma, lymphoma, intracranial tumors, and other cancer types [17, 19, 20].

Although it will take a tremendous effort from the research community to address many of these remaining challenges to “-omics” integration, there are useful methods currently available and no shortage of available “-omics” data. With the massive amounts of data that we have and are currently being generated, the challenge will now be to integrate these technologies to form a cohesive biological depiction of human disease, because only then can we claim to have considered all constituents collectively.

An enormous challenge is also the functional validation of the in silico findings in relevant living biological systems, as well as the development of adequate in vitro functional studies to keep up with the increasing throughput by which candidates for validation are generated. It is still crucial to explore functions of thousands of candidate genes, proteins, and metabolomics to ascertain their value as risk factors, as predictive factors for therapy response, and as therapeutic targets.

## 3. Integrative Omics in Personalized Medicine

While integrative analyses are important in all areas of genetics and genomics, they are especially important for mapping and obtaining a better understanding for the biology of drug response. There are a number of study design limitations in pharmacogenomics, pharmacoproteomics, and pharmacometabolomics that force the use of integrative approaches to make reliable discoveries. In most omics applications, replication is considered the gold standard—where potential associations are tested in one dataset and significant signals are validated in independent data. Obtaining and properly using these data are a particular challenge for drug response studies due to limitations in study design; for example, such studies are frequently nested within clinical trials, where sample sizes are extremely limited or treatment strategies are not completely comparable to the initial study. Because of this, replication samples are not routinely available and replication across omics levels is the only available option for reinforcing the initial discoveries.

Other opportunities for integrative approaches to address the needs in personalized medicine are in health monitoring. An example can be found in Chen et al. [21], where the authors develop an integrative personal omics profile (iPOP) analysis tool that tracks individual genomic, transcriptomic, proteomic, metabolomic, and autoantibody profiles [21]. This technology is successfully leveraged to identify healthy and diseased states for a single individual [21]. These approaches are in their infancy but provide great hope for the management and prevention of complex disease. Additional, integrative approaches specific for personalized medicine are thoroughly reviewed in Chen and Snyder [22].

## 4. Conclusions

A more fundamental understanding of the biological dynamics across omics datasets will enable us to better identify risk factors, refine disease diagnosis, predict therapeutic effects and prognosis, and identify new targets for therapy in personalized medicine. While the biological intuition of integrative “-omics” is clear, the real challenges are related to data integration, curation, and analysis. As we are moving towards an era in which the amount of data produced every year is increasing exponentially, methods to develop a deeper understanding of the biology of complex systems are crucial.
